# Human oral isolate *Lactobacillus fermentum* AGR1487 induces a pro-inflammatory response in germ-free rat colons

**DOI:** 10.1038/srep20318

**Published:** 2016-02-04

**Authors:** Rachel C. Anderson, Dulantha Ulluwishewa, Wayne Young, Leigh J. Ryan, Gemma Henderson, Marjolein Meijerink, Eva Maier, Jerry M. Wells, Nicole C. Roy

**Affiliations:** 1 Food Nutrition & Health Team, Food & Bio-based Products Group, AgResearch Grasslands, Palmerston North, New Zealand; 2 Riddet Institute, Massey University, Palmerston North, New Zealand; 3 Rumen Microbiology Team, Animal Health & Nutrition Group, AgResearch Grasslands, Palmerston North, New Zealand; 4 Host-Microbe Interactomics, Animal Sciences Group, Wageningen University, Wageningen, The Netherlands; 5 Gravida: National Centre for Growth and Development, The University of Auckland, Auckland, New Zealand

## Abstract

Lactobacilli are thought to be beneficial for human health, with lactobacilli-associated infections being confined to immune-compromised individuals. However, *Lactobacillus fermentum* AGR1487 negatively affects barrier integrity *in vitro* so we hypothesized that it caused a pro-inflammatory response in the host. We compared germ-free rats inoculated with AGR1487 to those inoculated with another *L. fermentum* strain, AGR1485, which does not affect *in vitro* barrier integrity. We showed that rats inoculated with AGR1487 had more inflammatory cells in their colon, higher levels of inflammatory biomarkers, and increased colonic gene expression of pro-inflammatory pathways. In addition, our *in vitro* studies showed that AGR1487 had a greater capacity to activate TLR signaling and induce pro-inflammatory cytokines in immune cells. This study indicates the potential of strains of the same species to differentially elicit inflammatory responses in the host and highlights the importance of strain characterization in probiotic approaches to treat inflammatory disorders.

A dysfunctional intestinal barrier or loss of epithelial barrier integrity leads to inflammatory responses in the mucosa due to signaling via innate pattern-recognition receptors such as the Toll-like receptors (TLRs) recognizing microbe-associated molecular patterns (MAMPS). If not resolved this leads to mucosal damage from host inflammation and a vicious cycle of barrier destruction and inflammation^1^,^2^,^3^,^4^. Despite the role of TLR activation in the induction of inflammatory responses, a ‘tonic’ level of constitutive TLR activation by commensal bacteria is considered to be crucial in the recovery from epithelial damage^5^. This was evident from studies in mice that are deficient in inhibitor of kappa B kinase gamma (IκB kinase-γ; NEMO), which develop spontaneous colitis due to the failure of nuclear factor kappa B (NF-κB) to induce epithelial repair and steady-state production of innate effector mechanisms in the intestine^6^. Additionally, TLR2 signaling has been implicated in tight junction regulation *in vivo* and *in vitro*^7^.

Lactobacilli are thought to be beneficial for human health, with many studies describing their use for treatment and prevention of immune and intestinal disorders *in vivo,* including allergic diseases, chronic inflammatory diseases and diarrhea^8^,^9^. The proposed mechanisms underlying the beneficial effects of lactobacilli on health include antagonism of pathogens, enhancement of intestinal epithelial barrier functions, and effects on immune cells and adaptive immunity depending on the probiotic strain^10^,^11^.

To date, there have been few reports of lactobacilli having an adverse effect on a healthy host. Lactobacilli are rarely associated with conditions such as endocarditis, bacteremia, and abscesses (less than 1 case per million individuals^12^); however, these generally occur in people with underlying health problems or immuno-compromised individuals^13^. A commonly used probiotic, *L. rhamnosus* GG, was isolated from patients with bacteremia^14^,^15^ and from a liver abscess^16^. However, despite the increased consumption of *L. rhamnosus* GG in probiotic foods, there has not been an increase in lactobacilli-associated bacteremia^17^. Therefore, it is likely that the cases of *L. rhamnosus* GG infection are a result of the susceptibility of the individuals involved (mostly immuno-compromised) as opposed to any inherent detrimental characteristics of the strain.

*Lactobacillus fermentum* AGR1487 may be the first example of a lactobacilli strain with inherent undesirable effects on its host, particularly in relation to intestinal function. The human oral isolate AGR1487 was classified as belonging to the *L. fermentum* species based on its 16S rRNA sequence^18^. *L. fermentum* is a commensal species found throughout the human gastrointestinal tract. Lactobacilli found in human faeces can originate from the mouth^19^,^20^, indicating that this oral isolate may reside in the intestines of the individual from whom it was isolated. AGR1487 was shown to increase intestinal epithelial barrier permeability *in vitro*, possibly by increasing the turnover of microtubules in the epithelial cells leading to tight junction disassembly^21^. In addition to reducing intestinal epithelial barrier integrity, the *in vitro* study also suggested that AGR1487 may have a pro-inflammatory effect^21^.

To test the hypothesis that AGR1487 induces a pro-inflammatory response in the host we compared it with another human oral isolate of *L. fermentum*, AGR1485, which does not alter epithelial barrier integrity *in vitro*^21^. The effects of AGR1485 and AGR1487 on TLR activation and dendritic cell function were investigated *in vitro* as well as their effects on the mucosa of mono-colonized germ-free rats. It was important to compare AGR1487 inoculated rats to those inoculated with a similar strain, not to un-inoculated rats, because the differences between germ-free rats and rats colonized by any bacterium are vast.

## Results

### AGR1487 is more a potent inducer of TLR signaling than AGR1485

**Figure 1:**
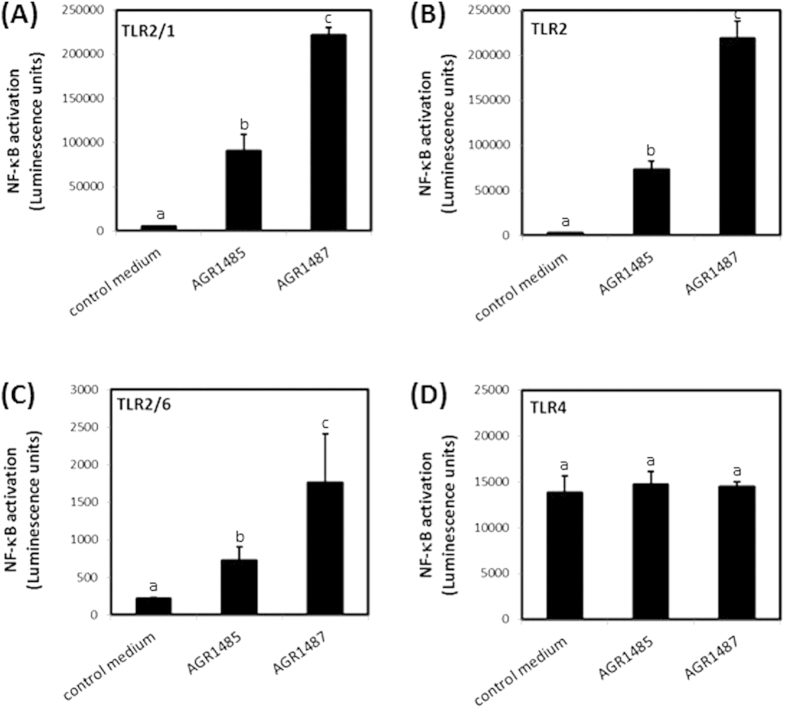
Effect of AGR1485 and AGR1487 on NF-κB activation via TLRs (A) 2/1, (**B**) 2, (**C**) 2/6 and (**D**) 4. HEK293 cells stably transfected with human TLRs and a reporter plasmid containing luciferase under the control of the human NF-κB promoter were exposed to the bacteria treatments (10 bacterial cells per HEK293 cell) for 4 hours and the resulting luminescence was measured. Values are the means +/− SEM; n = 4 per treatment. The means were significantly different if the lower case letters were not shared between treatments (*P* < 0.05).

The ability of the two *L. fermentum* strains to activate TLRs expressed in intestinal epithelial cells (i.e. 2/1, 2, 2/6 and 4) was measured. The HEK293 cell lines used expressed the human TLRs of interest and carried a luciferase reporter under control of a NF-κB responsive promoter. The different reporter cell lines responded only to the specific agonists for the TLRs that were expressed in these cells (Pam_2_CSK4 for TLR2 and TLR2/6; Pam_3_CSK4 for TLR2/1; lipopolysaccharide (LPS) for TLR4). As expected, AGR1485 and AGR1487 did not induce NF-κB in HEK293 cells carrying only the luciferase reporter under control of an NF-κB responsive promoter. In contrast, both strains induced NF-κB in HEK293 reporter cells expressing human TLRs 2/1, 2 and 2/6 (*P* < 0.05; Fig. 1A–C) compared to control medium, but not expressing TLR4 (Fig. 1D), showing they express microbe-associated molecular patterns (MAMPs) that bind to these receptors. The NF-κB reporter activity was 2.4-fold greater for strain AG1487 than AGR1485 for TLR2/1 and TLR2/6, and 3-fold greater for TLR2 (*P* < 0.05).

### AGR1487 has a greater capacity than AGR1485 to stimulate dendritic cell maturation and cytokine secretion

**Figure 2:**
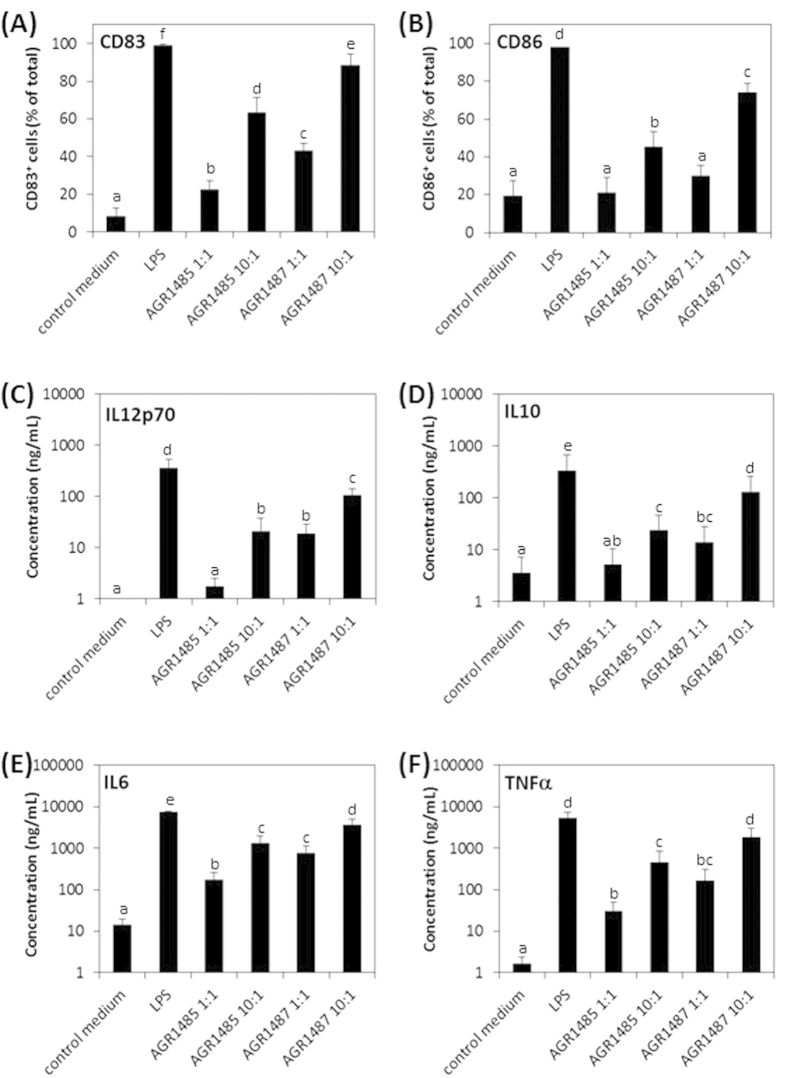
Effect of AGR1485 and AGR1487 on dendritic cell maturation and cytokine production. Human monocyte-derived immature dendritic cells were exposed to the bacteria treatments (1 or 10 bacterial cells per dendritic cell) for 48 hours. The resulting dendritic cell maturation markers (**A**) CD83 and (**B**) CD86, as well as the secretion of cytokines (**C**) IL12p70, (**D**) IL10, (**E**) IL6 and (**F**) TNFα, were measured. Values are the means +/− SEM; n = 5 donors per treatment. The means were significantly different if the lower case letters were not shared between treatments (P < 0.05).

Human monocyte-derived immature dendritic cells were stimulated with AGR1485 or AGR1487, and their maturation status and cytokine secretion profiles (IL12p70, IL10, IL6 and TNFα) were measured. As expected, the dendritic cells responded to LPS (1 μg/mL) by up-regulating expression of CD83, CD86, IL12p70, IL6, IL10, and TNFα compared to control medium (Fig. 2). The percentage of dendritic cells that expressed the maturation markers CD83 and CD86 after stimulation with AGR1487 or AGR1485 were dose dependent; a dose of ten bacterial cells per dendritic cell caused a greater percentage of cells to express markers of maturation compared to a dose of one bacterial cell per dendritic cell (*P* < 0.05; Fig. 2A,B). AGR1487 stimulation led to 20% more maturation of the dendritic cells compared to AGR1485 (*P* < 0.05). Similarly, the concentration of the cytokines secreted by the dendritic cells was dependent on the dose of the bacteria, and those stimulated with AGR1487 produced higher amounts of all measured cytokines compared to those stimulated with AGR1485 (Fig. 2C–F). For the lower treatment dose, the amount of pro-inflammatory IL12p70 secreted by dendritic cells in response to AGR1487 was ten-fold higher than that in response to AGR1485 (18.95 vs 1.77 ng/mL; *P* < 0.05); whereas, the amount of anti-inflammatory IL10 secreted was only 2.6-fold higher (13.84 vs 5.24 ng/mL; *P* < 0.05). Therefore, the IL10/IL12 ratio for AGR1487 was only 0.73, compared to 2.96 for AGR1485. This lower IL10/IL12 ratio, along with the higher levels of TNFα, indicates that AGR1487 induced a more pro-inflammatory response than AGR1485.

### AGR1485 and AGR1487 colonized the intestine of germ-free rats

In order to compare the effects of the strains *in vivo*, germ-free rats were inoculated with 10^9^ bacterial cells of either AGR1485 or AGR1487 and monitored for two weeks before sampling. There were no differences in daily feed intake (AGR1485: 17.4 +/− SEM 0.5 vs. AGR1487: 16.6 +/− SEM 0.5 g/day; *P* > 0.05) or overall weight gain (AGR1485: 34.4 +/− SEM 6.9 vs AGR1487: 40.8 +/− SEM 3.0 g/day; *P* > 0.05) between the treatment groups. Throughout the experiment all rats remained healthy (General Health Score of 5), with no visible differences in health status between the two treatment groups. The concentration of colony-forming bacterial cells present in the rat feces during the experimental period, and in the caecum and colon contents at the end of the experimental period, were 1000-fold higher for those inoculated with AGR1487 compared to AGR1485 (AGR1487: 10^8^ vs AGR1485: 10^5^ colony forming units (CFU)/g; *P* < 0.05). There was a smaller difference in the concentration of viable bacterial cells between the treatment groups in the ileum (AGR1487: 10^6^ vs AGR1485: 10^5^ CFU/g contents; *P* < 0.05) and there was no difference in the duodenum or jejunum (10^5^ CFU/g contents; *P* > 0.05).

### AGR1487 increased the number of macrophages and lymphocytes in the colon compared to AGR1485

**Figure 3:**
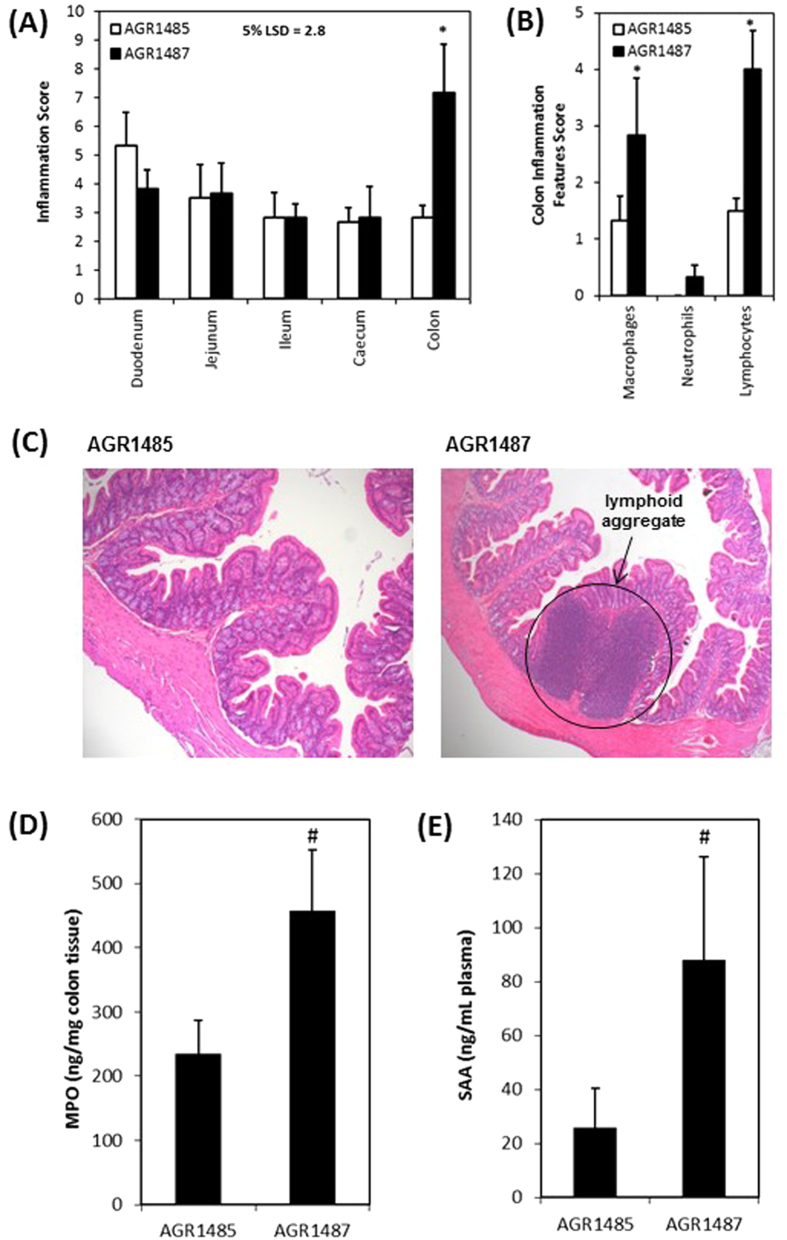
Measures of inflammation in intestinal samples from germ-free rats inoculated with AGR1485 or AGR1487. (**A**) Total inflammation scores for each intestinal tissue; (**B**) Individual inflammation scores for each feature in the colon; (**C**) Typical images of colon samples of rats inoculated with AGR1485 or AGR1487; (**D**) Concentration of myeloperoxidase (MPO) in colon tissue; (**E**) Concentration of serum amyloid A (SSA) in plasma samples. Values are the means +/− SEM; n = 6 per treatment. *P < 0.05 between treatments. ^#^*P* < 0.10 between treatments.

Histological analysis of the rat duodenum, jejunum, ileum, caecum and colon tissues was performed including evaluating the presence of immune cells (macrophages, lymphocytes and neutrophils) and physical damage (crypt hyperplasia, aberrant crypt, crypt injury, crypt loss, goblet cell loss, crypt abscess, sub-mucosal thickening, hyperchromatia and surface loss). Increased numbers of inflammatory cells were present in the colon of rats mono-associated with AGR1487 for two weeks than rats colonized with AGR1485 (*P* < 0.05; Fig. 3A), but there were no differences between treatment groups in the other intestinal tissues. There was a significant increase in the scores for macrophages and lymphocytes, indicating there were greater numbers of these cells, in the colon of AGR1487 colonized rats compared to the colon of AGR1485 colonized rats (*P* < 0.05; Fig. 3B). Higher numbers of neutrophils were counted in colon of AGR1487 colonized rats than AGR1485 colonized rats but these were not significant. Lymphoid cell aggregates resembling solitary intestinal lymphoid tissues (SILT), which develop after birth under the continuous exposure to commensals as well as potential pathogens were observed only in the AGR1587 colonized rats only (typical images of the colon are given in Fig. 3C). There were no differences between treatment groups in the physical damage score for any of the tissues (treatment × tissue effect *P* = 0.543).

### AGR1487 increased the concentration of biomarkers of inflammation compared to AGR1485

The concentrations of two biomarkers of inflammation were measured: myeloperoxidase (MPO), which is abundantly produced in neutrophils and to a lesser extent in macrophages and dendritic cells, and serum amyloid A (SAA), which is a plasma marker of acute phase inflammation. The average MPO concentration was approximately twice as high in the colon tissue of the rats mono-associated with AGR1487 compared to those mono-associated with AGR1485 (*P* = 0.07; Fig. 3D). Similarly, the average plasma SAA concentration was about 3.5 times higher in the rats mono-associated with AGR1487 compared to those mono-associated with AGR1485 (*P* = 0.08; Fig. 3E). In the AGR1485 group, four out of six rats had plasma SAA concentrations lower than the limit of detection (5.79 ng/mL); whereas, for the AGR1487 group, this was observed for only one rat. Multivariate analysis of the histology and biomarker measures of inflammation (macrophages, neutrophils, lymphocytes, MPO and SAA) showed there were differences in levels of inflammation markers between the two treatment groups (*P* = 0.03), indicating that AGR1487 elicits a stronger pro-inflammatory response *in vivo* than AGR1485.

### AGR1487 and AGR1485 had distinct effects on gene expression in the colon

**Figure 4:**
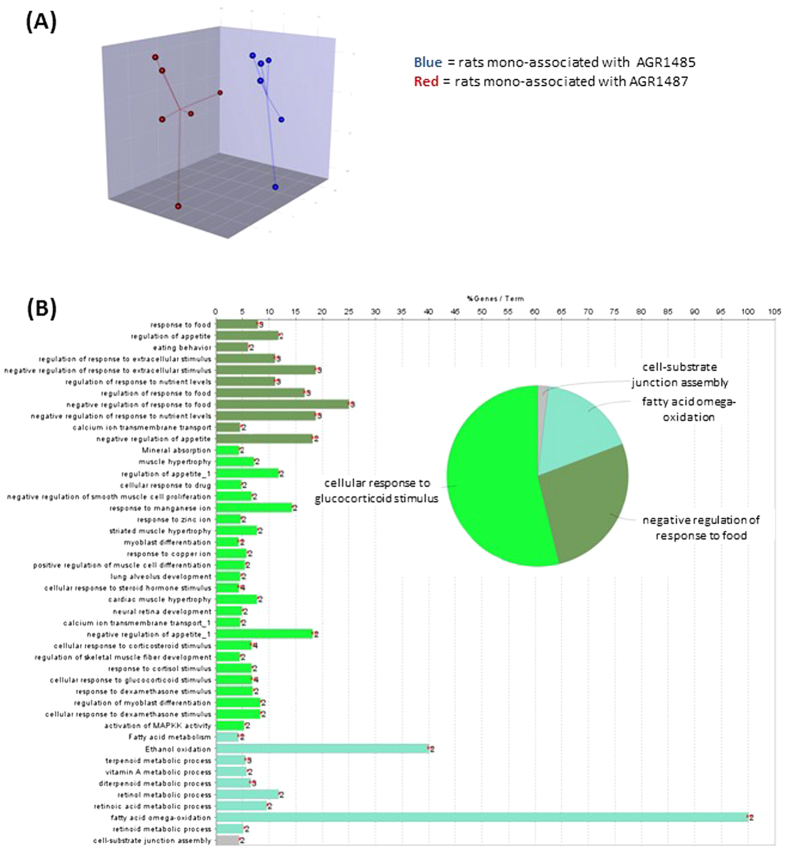
Analysis of gene expression of colon samples from rats mono-associated with AGR1485 versus AGR1487. (**A**) Plot showing the Partial Least Squares Discriminant Analysis (PLS-DA) of the gene expression profiles for genes showing the greatest variation in expression (top 5% coefficient of variation). The dots represent the individual samples in each treatment group. (**B**) Summary of the gene ontology biological processes that were significantly over-represented among genes differentially expressed between treatment groups. The bar graphs show the percentage of genes that were differentially expressed in the given biological process and the numbers at the end of the bars are the number of differentially expressed genes. Biological processes that are related are shown in the same color. The pie chart shows the proportion of the biological process that belong to each group.

Microarray analysis was carried out to compare the gene expression profiles in response to the two bacterial treatments in the rat colon. The data have been deposited in NCBI’s Gene Expression Omnibus and are accessible through GEO Series accession number GSE58777. AGR1485 and AGR1487 had distinct effects on gene expression rat colon, as illustrated by the separation of the treatment groups in the partial least squares discriminant analysis (PLS-DA) of the gene expression profiles (Fig. 4A). Sixty-nine genes were differentially expressed (fold-change >1.5; *P* < 0.01) between the AGR1485 and AGR1487 treatment groups (Supplementary Table S1).

### AGR1487 altered expression of small molecule transport and metabolism, and nervous system development pathways in the colon

The biological roles of the colon genes that were differentially expressed between treatments were examined using a range of analyses. Over-representation analysis showed that differentially expressed genes between treatment groups were over-represented in 46 gene ontology biological processes, with 54% of those being related to ‘cellular response to glucocorticoid stimulus’ (Fig. 4B and Supplementary Figure S1). Other processes were associated with ‘negative regulation of response to food’, ‘fatty acid omega-oxidation’ and ‘cell-substrate junction assembly’. Of the top 10 IPA canonical pathways enriched with differentially expressed genes in the colon, four and two pathways were associated with small molecule degradation and biosynthesis, respectively (Table 1). The others were associated with cellular proliferation, synapse transmission and immune signaling, including NF-κB signalling. Based on the changes in gene expression, IPA predicted two upstream regulators to be altered in the colon of rats mono-associated with AGR1487 compared to those associated with AGR1485; insulin 1 was predicted to be inhibited (activation score −2.219; *P* = 0.002) and IL6 was predicted to be activated (activation score 2.384; *P* = 0.012). Overall the gene expression analysis supports the idea that AGR1487 induced a pro-inflammatory response.

## Discussion

In agreement with the hypothesis that AGR1487 would be more pro-inflammatory than AGR1485, it was found that colon of rats colonized with AGR1487 had greater inflammatory cell scores, including increased numbers of macrophages and lymphocytes, than the colon of rats colonized with AGR1485. The AGR1487 treated rats also had increased amounts of colonic MPO and plasma SAA, both biomarkers of inflammation. Although the differences in MPO and SAA between treatment groups were a trend (*P* < 0.1), this was likely due to the limited number of rats (6 per treatment) that were feasible to house in the germ-free isolators.

A greater number of AGR1487 bacterial cells were present in the in the rat ileum, caecum and colon than AGR1485. This was expected based on previous *in vitro* studies showing AGR1487 had increased tolerance to gastrointestinal conditions (bile salts and low pH)^22^. It is unlikely that the increased pro-inflammatory response to AGR1487 in the colon was solely due to the fact that more bacterial cells were present because histological signs of inflammation were not observed in the caecum where AGR1487 was present in greater numbers compared to the colon. Localization of inflammation to the colon is a known phenomenon. For example, mice lacking Muc2 and secreted mucus develop colitis but no pathology in the ileum due to the lower abundance of bacteria in the small intestine and induction of the IL-22 pathway of genes regulating barrier function and innate defences^23^.

Analysis of the colon tissue gene expression supported the histology and biomarker data suggesting an increased pro-inflammatory response in the presence of AGR1487. For example, there was increased expression of genes involved in ‘CREB phosphorylation through the activation of Ras’, which is known to activate p38 MAPK signaling^24^, in rats colonized with AGR1487. Similarly, genes involved in ‘NF-κB Signalling’ were differentially expressed between treatment groups as expected based on the TLR activation assay results. In addition, IL6, which is thought to play a crucial role in intestinal inflammatory disorders^25^,^26^, was predicted to be ‘activated’ in the colon of rats inoculated with AGR1487 (compared to AGR1485). This cytokine was also secreted in higher amounts by dendritic cells stimulated with AGR1487 compared to AGR1485 *in vitro.*

Furthermore, the majority of colon genes differentially expressed between treatments were over-represented in the ‘cellular response to glucocorticoid stimulus’ biological process. This analysis showed that the pathway was changed between treatments, but not the direction of the change by a specific treatment. Glucocorticoids are inhibitors of inflammation and are commonly used to treat inflammatory conditions by balancing out pro-inflammatory cytokine production^27^. Thus the observed differential gene expression could potentially be indicating differences in inflammatory responses of AGR1485 and AGR1487 in the rat colon.

The TLR signaling and dendritic cell phenotype assays support the claim that AGR1487 elicits a pro-inflammatory response in the host. The differential capacities of the strains to elicit inflammatory responses is likely to be due to differences in their surface structures as antibiotics were included in the tissue culture medium to prevent growth and production of metabolites. AGR1487 induced greater activation of both TLR2/1 and TLR2/6 than AGR1485. This does not confirm that AGR1487 has a negative effect on the host; in fact, in the intestine TLR2 activation is necessary for epithelial repair^5^. The association between TLR2 activation, p38 MAPK signaling and tight junction integrity is not fully understood. Stimulation of the TLR2 pathway leads to activation of specific protein kinase C isoforms *in vitro*, ultimately leading to the sealing of tight junctions and an increase in TEER^28^ or prevention of tight junction disruption^7^. Although AGR1487 activated TLR2, the strain disrupted tight junctions and caused a decrease in TEER of epithelial cell monolayers^21^. Caco-2 cells treated with AGR1487 had higher expression levels of genes involved in the p38 MAPK signalling pathway, which leads to tight junction disruption^29^. Given that p38 MAPK signalling is activated by TLR2 stimulation, this highlights how TLR2 likely plays multiple roles depending on the stimulus and co-receptor.

Collectively the data indicate that AGR1487 induces a pro-inflammatory response but it is unclear whether this is detrimental to the host or part of the natural intestinal maturation process. The General Health Score of the rats mono-associated with AGR1487 did not decrease over the course of the study. This may be due to the short duration of the study (2 weeks) not being long enough for indicators of poor health to be observed, or it may be that the low level inflammation alone is not enough to cause observable deteriorations in health status. The lymphoid structures in the colonic mucosa of AGR1487 colonized rats resembled SILT, and there was no associated crypt damage that is typical of colitis. The development of SILT structures in the colon has only recently been reported and was shown to be dependent on TLR and MyD88 signaling^30^, which were activated by AGR1487. SILT development is dependent on lymphotoxin a, which was not differentially expressed between the treatment groups; however, gene expression does not always correlate with protein expression.

It is possible that the effect of AGR1487 is dependent on the genotype and phenotype of the host. As an example of this, the segmented filamentous bacteria (SFB) are thought to play a critical role in appropriate intestinal immune maturation by inducing a multifaceted immune response^31^,^32^,^33^. However, SFB are also associated with auto-immune responses in genetically-susceptible hosts^34^,^35^. One of the limitations of the present study is that the effect of the bacteria were tested in isolation with one rat genetic background. Future studies could investigate whether AGR1487 causes a similar low level inflammatory response in rodents colonized with specific-pathogen-free microbiota and with different genetic backgrounds, or germ-free mice colonized with a human microbiota.

In conclusion, this research showed that AGR1487 elicits a stronger pro-inflammatory response than AGR1485 in the colon of mono-colonized germ-free rats. These phenotypic changes were supported by transcriptomic analysis of rat colon tissue which indicated that, compared to AGR1485, AGR1487 increased the rat colon expression of gene pathways involved in various inflammation processes including those related to the cytokine IL6. In addition, the *in vitro* studies gave further insights into the mechanism of the adverse effect of AGR1487. AGR1487 has greater capacity to activate TLR signaling, which reinforced previous data showing that the intestinal epithelial genes involved in MAPK signaling were up-regulated by this bacterium. Furthermore, compared to AGR1485, AGR1487 induced more dendritic cells to mature resulting in higher cytokine production, including higher pro-inflammatory TNFα production and a lower IL10 to IL12 ratio. To build on this knowledge, current research is comparing the genome sequences of the two strains (AGR1485 and AGR1487) to identify differences that may be responsible for the contrasting effects. This study indicates the potential of strains of the same species to differentially elicit inflammatory responses in the host and highlights the importance of strain characterization in probiotic approaches treat inflammatory disorders. In the long term this research will increase the understanding of the complex host-microbe interactions in the human intestine that influence inflammatory responses in the host.

## Methods

### Bacterial strains

The two bacterial strains used in this research were isolated from saliva swabs of adult human volunteers as previously described^18^. Ethical approval from the New Zealand Health and Disability Committee was not required due to the non-invasive nature of the sampling and the healthy status of the volunteers. Written consent for collection and use of the samples for research purposes was obtained from the volunteers. AGR1485 was isolated from a healthy individual and AGR1487 was isolated from a then apparently healthy individual who was later diagnosed with inflammatory bowel disease (IBD). For the experiments, the two bacterial strains were inoculated from frozen stocks onto de Man, Rogosa and Sharpe (MRS) agar (Difco) and grown for 48 hours in 5% CO_2_ at 37 °C. Single colonies were inoculated into primary MRS broths and grown for 24 hours in 5% CO_2_ at 37 °C, before 100 μL of the primary broth was inoculated into the secondary broth and grown overnight in 5% CO_2_ at 37 °C to ensure the bacteria were in stationary phase.

### TLR activation assay

The human embryonic kidney cell line, HEK293, was stably transfected to express human TLRs (TLR2, TLR2/1, TLR2/6 or TLR4) and to carry a reporter plasmid containing the luciferase gene under the control of the human NF-κB promoter (pNiFty2-Luc, Invivogen). The HEK293-TLR-Luc cell lines were seeded into transparent-bottomed black 96-well plates at a density of 6 × 10^4^ cells/well in 200 μL DMEM glutamax + Glucose (Invitrogen) containing 10% v/v heat inactivated fetal bovine serum (FBS), 0.5% v/v penicillin-streptomycin solution (Invitrogen, Breda, the Netherlands), 10 μg/mL blasticidin (Invivogen) and 250 μg/mL zeocin (Invivogen), and incubated at 37 °C with 5% CO_2_ overnight until approximately 80% confluence. The medium was removed and 200 μL of the following stimulations were added (n = 4 per treatment): control media, TLR-ligand positive control (5 ng/mL Pam_2_CSK4 for TLR2 and TLR2/6; 5 ng/mL Pam_3_CSK4 for TLR2/1 and 25 ng/mL LPS for TLR4), AGR1485 and AGR1487. Ten bacterial cells were added per HEK293-TLR-Luc cell. The HEK293-TLR-Luc cells were incubated with the treatments for 4 hours, then 120 μL of the solution was removed and 100 μL of Bright-Glo substrate (Promega) was added. The plate was shaken for 5 minutes at 500 rpm, then the luminescence was measured (top read; 750 ms integration time). HEK293 cells transfected with only the pNiFty2-Luc plasmid did not respond to Pam_2_CSK4, Pam_3_CSK4 nor LPS, demonstrating the dependency of NF-κB activation on co-expression of the different receptors.

### Dendritic cell maturation and cytokine production assays

The study was approved by Wageningen University Ethical Committee and was performed according to the principles of the Declaration of Helsinki. Buffy coats from five individual blood donors were obtained from the Sanquin Blood bank Nijmegen (The Netherlands). Informed consent was obtained before the sample collection. Human peripheral blood mononuclear cells (PBMCs) were isolated from the buffy coat using a Ficoll density centrifugation, followed by CD14^+^ monocytes were separation using antibody coated microbeads (Miltenyi Biotec) as previously described^36^. To generate immature dendritic cells, approximately 10^6^ CD14^+^ cells/well were cultivated in RPMI 1640 containing 10% FBS gold (PAA), 1% penicillin-streptomycin, 50 ng/mL IL-4 (R&D systems) and 50 ng/mL granulocyte macrophage-colony stimulating factor (GM-CSF; R&D systems) in a 24-well plate at 37 °C in 5% CO_2_ for 6 days. On days 3 and 6, half of the medium was refreshed. On day 6 the immature dendritic cells were treated with control media, 1 μg/mL LPS (positive control), AGR1485 1:1 (1 bacteria cell per dendritic cell), AGR1485 10:1 (10 bacteria cells per dendritic cell), AGR1487 1:1 or AGR1487 10:1. The treated dendritic cells were incubated at 37 °C in 5% CO_2_ for 48 hours. After treatment the cells and culture media were harvested for analysis.

To assess the maturation status of the dendritic cells, the cells were stained with specific monoclonal antibodies to CD83, CD86 or their isotype-matched controls (BD Biosciences) for 30 min on ice, washed and analyzed by flow cytometry (FACSCanto II, BD Biosciences). The flow cytometry data were analyzed using the BD FACSDiva software. To assess the production of cytokines by the stimulated dendritic cells, the cell supernatants were analyzed for the presence of IL12p70, IL10, IL6 and TNFα using a cytometric bead-based immunoassay that enables multiplex measurements of soluble cytokines in the same sample, according to the manufacturer’s protocol (BD Biosciences). The cytokine concentrations were calculated using the BD FCAP software.

### Mono-associated rat experiment

This rodent experiment was approved by the AgResearch Grasslands Animal Ethics Committee (Palmerston North, New Zealand; AEC 12504) in accordance with the New Zealand Animal Welfare Act 1999. Twelve female germ-free JCL:Wistar rats between 7 and 8 weeks of age were obtained from Japan SCL Inc (Tokyo, Japan) and transported to New Zealand in germ-free transport containers. On arrival the rats were transferred to individual cages in two separate sterile isolators (Semi-Rigid Isolator, purchased from Charles River Laboratories, manufactured by Britz & Co) using standard operating procedures (n = 6 per isolator). The rats underwent an adaptation period of 7 days prior to inoculation via oral gavage. Each rat was given approximately 10^9^ bacterial cells of either AGR1485 or AGR1487.

Throughout the experiment the rats were fed sterile standard rat chow (AIN-76a, Research Diets Inc) and water *ad libitum*. All consumables and equipment were sterilized prior to entering the isolators; food and most husbandry items were irradiated and water was autoclaved. Each day, rats were checked for the presence of loose stools or blood in feces (an indication of intestinal inflammation) and the General Health Score (commonly used criteria for rating rat wellness in a range from 1 (very ill) to 5 (healthy)) was recorded^37^. The rats were weighed three times a week. For each rat the overall weight gain was calculated using the following equation: final live weight (14 days after inoculation) – initial live weight (day of inoculation). Additionally, mean daily food intake was calculated as the total food intake/number of days.

Fourteen days after inoculation the rats were removed from the isolators, and euthanized by CO_2_ asphyxiation and cervical dislocation. Intestinal tissue samples (duodenum, jejunum, ileum, caecum and colon) were immediately collected post mortem and rapidly frozen in liquid nitrogen, or fixed in formalin and stored at room temperature, until analysis. Intestinal contents samples were processed within 1 hour of collection.

### Fecal and intestinal contents viable bacteria concentrations

Fecal pellets were collected 3, 4, 6, 8, 10 and 13 days after inoculation with the test bacterial strains. Duodenum, jejunum, ileum, caecum, and colon contents were collected during animal sampling 14 days after inoculation with the test bacterial strains. The fecal pellets and intestinal contents were suspended in sterile PBS solution (Gibco) at a dilution of 1/10 (w/v) and mixed using a vortex. Duplicate ten-fold serial dilutions of the bacterial solutions were made in 96-well plates for each sample. Three 20 μL spots of each dilution were pipetted onto MRS agar, allowed to dry and then incubated overnight in 5% CO_2_ at 37 °C. Spots with between 10 and 100 colonies were counted and the colony forming units (CFU) were calculated.

### Histological scoring of intestinal tissues

For each intestinal tissue sample (duodenum, jejunum, ileum, caecum, and colon) histological signs of damage and inflammation were assessed. The tissue samples were fixed in 10% formalin at tissue sampling and stored at room temperature. The samples were embedded in paraffin and sectioned to 5 μm, and then stained with haematoxylin and eosin for examination. The stained sections were assessed for signs of immune cell infiltration (macrophages, neutrophils, and lymphocytes) and physical damage (crypt hyperplasia, aberrant crypts, crypt injury, crypt loss, goblet cell loss, crypt abscess, lymphoid aggregates, sub-mucosal thickening, hyperchromasia, and surface loss) by an individual blinded to the treatments. Each parameter was scored out of 10 (1=low; 10=high).

### Biomarker analysis

Colonic MPO and plasma SAA concentrations were determined using ELISA kits (CUSABIO; CSB-E08722r and CSB-E08590r) according to the manufacturer’s instructions. The colon samples were homogenized in PBS buffer at a concentration of 100 mg tissue/mL. Two freeze-thaw cycles were performed to break the cell membranes, then the homogenates were centrifuged at 5000 × *g*. The supernatant was removed and used for the assay. The detection limits were calculated to be 2.88 ng/mL for MPO and 5.97 ng/mL for SAA (mean plus two standard deviations of blanks).

### Whole genome expression analysis

Colon samples were homogenized in buffer RLT (Qiagen, California, USA) and total RNA was extracted using an AllPrep DNA/RNA/Protein Mini Kit (Qiagen) as per the manufacturer’s protocol. The RNA quantity and purity was determined using a Nanodrop ND-1000 spectrophotometer (Nanodrop Technologies) and an RNA 6000 NanoLabChip kit with an Agilent 2100 Bioanalyzer (Agilent Technologies). All samples had an OD_260nm_/OD_280nm_ ratio greater than 2.0, a Bioanalyzer 28 s/18 s peak ratio greater than 1.5, and a RNA integrity number (RIN) greater than 8. Global gene expression profiles of the tissue samples were analyzed using Agilent Technologies 44k whole rat genome oligonucleotide arrays according to the manufacturer’s instructions (Two-Color Microarray-Based Gene Expression Analysis (Quick Amp Labeling), Version 5.7, March 2008, Agilent Technologies). Samples were hybridized to the microarrays using a reference design, where samples labelled with Cy3 were competitively hybridized with a reference RNA labelled with Cy5. The microarray slides were scanned using a DNA Microarray Scanner G2565CA (Agilent Technologies) according to the manufacturer’s instructions. Differentially expressed genes between the two treatment groups were identified by an Empirical Bayes modified T-statistic using the limma package (version 3.18.13, Smyth, 2004) in R (version 3.0.1; R Foundation for Statistical Computing, Vienna, Austria). Genes that exhibited a log2 fold change >0.58 (representing a fold change of approximately 1.5 time) and a P < 0.01 were determined to be differentially expressed. Partial least squares discriminant analysis (PLS-DA) of genes showing the greatest variation in expression (top 5% coefficient of variation) was performed using the mixOmics package (version 5.0-1) for R. Over-representation analysis of differentially expressed genes were performed using the ClueGO application (version 2.1.1) in Cytoscape (version 3.1). In addition, differentially expressed genes were clustered into functional groups and pathways using Ingenuity Pathway Analysis (IPA version 7.1; Ingenuity Systems Inc., Redwood City, CA, USA; www.ingenuity.com). Fisher’s exact test was used to calculate a P-value determining the probability that the association between the differentially expressed genes and the canonical pathway is explained by chance alone. The IPA transcription factor analysis identified transcription factors that could explain observed gene expression changes.

### Statistical analysis

All of the statistical analyses, other than that gene expression analysis, were completed using Genstat (v15.3). For the majority of the experiments, treatments were compared using a one-way ANOVA. The TLR assay luminescence values and the dendritic cell secreted cytokine concentrations, and the SAA concentrations were transformed (log(x + 1)) prior to analysis. For the feces and intestinal contents bacterial cell concentrations, and the histological analysis, treatments were compared using a two-way ANOVA (treatment and day as parameters for the feces samples, and treatment and tissue as parameters for the intestinal contents and histology samples). The macrophage, neutrophil and lymphocyte histology scores and the MPO and SAA biomarker concentrations were analysis collectively using MANOVA. Statistical difference was declared between two treatments where the difference in means was greater than the LSD at 5% and a trend was declared where the difference in means was greater than the LSD at 10%.

## Additional Information

**How to cite this article**: Anderson, R. C. *et al.* Human oral isolate *Lactobacillus fermentum* AGR1487 induces a pro-inflammatory response in germ-free rat colons. *Sci. Rep.* **6**, 20318; doi: 10.1038/srep20318 (2016).

## Supplementary Material

Supplementary Information

## Figures and Tables

**Table 1:** The top ten Ingenuity Pathway Analysis (IPA) ‘Canonical Pathways’ enriched with differentially expressed genes in the colon of rats mono-associated with AGR1487 compared to rats mono-associated with AGR1485.

IPA Canonical Pathways	P-value	Ratio
RAR Activation	0.002	0.023
Retinoate Biosynthesis I	0.004	0.061
Ethanol Degradation II	0.004	0.061
Noradrenaline and Adrenaline Degradation	0.005	0.057
S-adenosyl-L-methionine Biosynthesis	0.009	0.333
EGF Signalling	0.011	0.036
Arginine Degradation I (Arginase Pathway)	0.013	0.250
Serotonin Degradation	0.013	0.033
NF-κB Signalling	0.014	0.017
Agrin Interactions at Neuromuscular Junction	0.017	0.029

The ratio indicates the number of differentially expressed genes that map to the pathway divided by the total number of genes that map to the canonical pathway.

## References

[b1] Fasano A. Zonulin and its regulation of intestinal barrier function: The biological door to inflammation, autoimmunity, and cancer. Physiol. Rev. 91, 151–175 (2011). 21248165 10.1152/physrev.00003.2008

[b2] Hermiston M. L. & Gordon J. I. Inflammatory bowel disease and adenomas in mice expressing a dominant negative N-cadherin. Science 270, 1203–1207 (1995). 7502046 10.1126/science.270.5239.1203

[b3] Welz P. S. *et al.* FADD prevents RIP3-mediated epithelial cell necrosis and chronic intestinal inflammation. Nature 477, 330–334 (2011). 21804564 10.1038/nature10273

[b4] Kaser A. *et al.* XBP1 links ER stress to intestinal inflammation and confers genetic risk for human inflammatory bowel disease. Cell 134, 743–756 (2008). 18775308 10.1016/j.cell.2008.07.021 PMC2586148

[b5] Rakoff-Nahoum S., Paglino J., Eslami-Varzaneh F., Edberg S. & Medzhitov R. Recognition of commensal microflora by toll-like receptors is required for intestinal homeostasis. Cell 118, 229–241 (2004). 15260992 10.1016/j.cell.2004.07.002

[b6] Nenci A. *et al.* Epithelial NEMO links innate immunity to chronic intestinal inflammation. Nature 446, 557–561 (2007). 17361131 10.1038/nature05698

[b7] Karczewski J. *et al.* Regulation of human epithelial tight junction proteins by Lactobacillus plantarum *in vivo* and protective effects on the epithelial barrier. American Journal of Physiology. Gastrointestinal and Liver Physiology 298, G851–G859 (2010). 20224007 10.1152/ajpgi.00327.2009

[b8] Rijkers G. T. *et al.* Guidance for substantiating the evidence for beneficial effects of probiotics: current status and recommendations for future research. J. Nutr. 140, 671S–676S (2010). 20130080 10.3945/jn.109.113779

[b9] Kalliomäki M. *et al.* Guidance for Substantiating the Evidence for Beneficial Effects of Probiotics: Prevention and Management of Allergic Diseases by Probiotics1–3. The Journal of Nutrition 140, 713S–721S (2010). 20130079 10.3945/jn.109.113761

[b10] van Baarlen P., Wells J. M. & Kleerebezem M. Regulation of intestinal homeostasis and immunity with probiotic lactobacilli. Trends Immunol 34, 208–215 (2013). 23485516 10.1016/j.it.2013.01.005

[b11] Ulluwishewa D. *et al.* Regulation of tight junction permeability by intestinal bacteria and dietary components. J. Nutr. 141, 769–776 (2011). 21430248 10.3945/jn.110.135657

[b12] Borriello S. P. *et al.* Safety of probiotics that contain lactobacilli or bifidobacteria. Clin. Infect. Dis. 36, 775–780 (2003). 12627362 10.1086/368080

[b13] Besselink M. G. *et al.* Probiotic prophylaxis in predicted severe acute pancreatitis: a randomised, double-blind, placebo-controlled trial. Lancet 371, 651–659 (2008). 18279948 10.1016/S0140-6736(08)60207-X

[b14] Salminen M. K. *et al.* Lactobacillus bacteremia, clinical significance, and patient outcome, with special focus on probiotic L. rhamnosus GG. Clin. Infect. Dis. 38, 62–69 (2004). 14679449 10.1086/380455

[b15] De Groote M. A., Frank D. N., Dowell E., Glode M. P. & Pace N. R. Lactobacillus rhamnosus GG bacteremia associated with probiotic use in a child with short gut syndrome. Pediatr. Infect. Dis. J. 24, 278–280 (2005). 15750472 10.1097/01.inf.0000154588.79356.e6

[b16] Rautio M. *et al.* Liver abscess due to a Lactobacillus rhamnosus strain indistinguishable from L. rhamnosus strain GG. Clin. Infect. Dis. 28, 1159–1160 (1999). 10452653 10.1086/514766

[b17] Salminen M. K. *et al.* Lactobacillus bacteremia during a rapid increase in probiotic use of Lactobacillus rhamnosus GG in Finland. Clin. Infect. Dis. 35 (2002). 10.1086/342912 12410474

[b18] Anderson R. C., Cookson A. L., Kelly W. J., McNabb W. C. & Roy N. C. *Lactobacillus plantarum* DSM 2648 is a potential probiotic that enhances intestinal barrier integrity. FEMS Microbiol. Lett. 309, 184–192 (2010). 20618863 10.1111/j.1574-6968.2010.02038.x

[b19] Dal Bello F. & Hertel C. Oral cavity as natural reservoir for intestinal lactobacilli. Syst. Appl. Microbiol. 29, 69–76 (2006). 16423658 10.1016/j.syapm.2005.07.002

[b20] Maukonen J., Matto J., Suihko M. L. & Saarela M. Intra-individual diversity and similarity of salivary and faecal microbiota. J. Med. Microbiol. 57, 1560–1568 (2008). 19018030 10.1099/jmm.0.47352-0

[b21] Anderson R. C. *et al.* Human oral isolate Lactobacillus fermentum AGR1487 reduces intestinal barrier integrity by increasing the turnover of microtubules in Caco-2 cells. PLoS ONE 8, e78774 (2013). 24244356 10.1371/journal.pone.0078774 PMC3828418

[b22] Sengupta R. *et al.* Lactobacillus fermentum AGR1487 cell surface structures and supernatant increase paracellular permeability through different pathways. MicrobiologyOpen 4, 541–552 (2015). 25943073 10.1002/mbo3.260 PMC4554451

[b23] Sovran B. *et al.* IL-22-STAT3 pathway plays a key role in the maintenance of ileal homeostasis in mice lacking secreted mucus barrier. Inflamm. Bowel Dis. 21, 531–542 (2015). 25636123 10.1097/MIB.0000000000000319

[b24] McDermott E. P. & O’Neill L. A. Ras participates in the activation of p38 MAPK by interleukin-1 by associating with IRAK, IRAK2, TRAF6, and TAK-1. J. Biol. Chem. 277, 7808–7815 (2002). 11744690 10.1074/jbc.M108133200

[b25] Yamamoto M., Yoshizaki K., Kishimoto T. & Ito H. IL-6 is required for the development of Th1 cell-mediated murine colitis. J. Immunol. 164, 4878–4882 (2000). 10779797 10.4049/jimmunol.164.9.4878

[b26] Mitsuyama K., Sata M. & Rose-John S. Interleukin-6 trans-signaling in inflammatory bowel disease. Cytokine Growth Factor Rev. 17, 451–461 (2006). 17045835 10.1016/j.cytogfr.2006.09.003

[b27] Dejager L., Vandevyver S., Petta I. & Libert C. Dominance of the strongest: inflammatory cytokines versus glucocorticoids. Cytokine Growth Factor Rev. 25, 21–33 (2014). 24412262 10.1016/j.cytogfr.2013.12.006

[b28] Cario E., Gerken G. & Podolsky D., K. Toll-like receptor 2 enhances ZO-1-associated intestinal epithelial barrier integrity via protein kinase C. Gastroenterology 127, 224–238 (2004). 15236188 10.1053/j.gastro.2004.04.015

[b29] Costantini T. W. *et al.* Role of p38 MAPK in burn-induced intestinal barrier breakdown. J. Surg. Res. 156, 64–69 (2009). 19577248 10.1016/j.jss.2009.03.066 PMC4251589

[b30] Baptista A. P. *et al.* Colonic patch and colonic SILT development are independent and differentially regulated events. Mucosal Immunol. 6, 511–521 (2013). 22990625 10.1038/mi.2012.90 PMC3570605

[b31] Ivanov I. I. *et al.* Induction of intestinal Th17 cells by segmented filamentous bacteria. Cell 139, 485–498 (2009). 19836068 10.1016/j.cell.2009.09.033 PMC2796826

[b32] Chung H. *et al.* Gut immune maturation depends on colonization with a host-specific microbiota. Cell 149, 1578–1593 (2012). 22726443 10.1016/j.cell.2012.04.037 PMC3442780

[b33] Klaasen H. L. *et al.* Apathogenic, intestinal, segmented, filamentous bacteria stimulate the mucosal immune system of mice. Infect. Immun. 61, 303–306 (1993). 8418051 10.1128/iai.61.1.303-306.1993 PMC302719

[b34] Lee Y. K., Menezes J. S., Umesaki Y. & Mazmanian S. K. Proinflammatory T-cell responses to gut microbiota promote experimental autoimmune encephalomyelitis. Proc. Natl. Acad. Sci. USA 108 Suppl 1, 4615–4622 (2011). 20660719 10.1073/pnas.1000082107 PMC3063590

[b35] Wu H. J. *et al.* Gut-residing segmented filamentous bacteria drive autoimmune arthritis via T helper 17 cells. Immunity 32, 815–827 (2010). 20620945 10.1016/j.immuni.2010.06.001 PMC2904693

[b36] Meijerink M., Ulluwishewa D., Anderson R. C. & Wells J. M. Cryopreservation of monocytes or differentiated immature DCs leads to an altered cytokine response to TLR agonists and microbial stimulation. J. Immunol. Methods 373, 136–142 (2011). 21878338 10.1016/j.jim.2011.08.010

[b37] Gill H. S., Shu Q., Lin H., Rutherfurd K. J. & Cross M. L. Protection against translocating *Salmonella typhimurium* infection in mice by feeding the immuno-enhancing probiotic *Lactobacillus rhamnosus* strain HN001. Med Microbiol Immunol (Berl) 190, 97–104. (2001). 11827205 10.1007/s004300100095

