# Supplementary material for: Human oral isolate Lactobacillus fermentum AGR1487 induces a pro-inflammatory response in germ-free rat colons
Source: Sci Rep. 2016 Feb 4;6:20318. doi: 10.1038/srep20318 (PMC4740858; doi:10.1038/srep20318)

Human oral isolate *Lactobacillus fermentum* AGR1487 induces a pro-inflammatory response in germ-free rat colons

Rachel C. Anderson, Dulantha Ulluwishewa, Wayne Young' Leigh J. Ryan, Gemma Henderson, Marjolein Meijerink, Eva Maier, Jerry M. Wells & Nicole C. Roy

SUPPLEMENTARY TABLE S1 - Genes differentially expressed in the colon of rats mono-associated with *L. fermentum* AGR1485 versus *L. fermentum* AGR1487

Gene Name	Agilent Probe ID	Systematic Name	log Fold Change	Average expression	adj.P.Val	Description
Pyy	A_44_P116446	NM_001034080	1.3132484	13.34915368	6.15E-06	peptide YY precursor [Source:RefSeq peptide;Acc:NP_001029252]
Cck	A_44_P119019	NM_012829	1.53014547	9.656087548	1.24E-05	Cholecystokinin Cholecystokinin-39 Cholecystokinin-33 Cholecystokinin-22 Cholecystokinin-12 Cholecystokinin-8 [Source:UniProtKB/Swiss-Prot;Acc:P01355]
Tfrc	A_44_P214811	NM_022712	0.64889699	11.82287026	6.85E-05	transferrin receptor protein 1 [Source:RefSeq peptide;Acc:NP_073203]
Pappa	A_43_P22638	NM_001107939	-0.7778101	7.6707132	0.00015113	pappalysin-1 [Source:RefSeq peptide;Acc:NP_001101409]
Arg1	A_42_P758222	NM_017134	0.89613947	9.638474497	0.000163653	Arginase-1 [Source:UniProtKB/Swiss-Prot;Acc:P07824]
Col4a5	A_44_P105448	ENSRNOT00000025677	-0.7661855	8.870191008	0.000223523	Unknown
Mettl20	A_44_P273885	NM_198772	-0.6066434	8.931887671	0.000252219	Methyltransferase-like protein 20 [Source:UniProtKB/Swiss-Prot;Acc:Q6P7Q0]
Slc39a8	A_43_P18495	NM_001011952	-0.6354258	9.180128337	0.000472113	Zinc transporter ZIP8 [Source:UniProtKB/Swiss-Prot;Acc:Q5FVQ0]
Krt23	A_44_P1043278	TC590266	0.68482994	9.23241382	0.000505858	keratin, type I cytoskeletal 23 [Source:RefSeq peptide;Acc:NP_001008753]
Pum2	A_44_P1042901	NM_001106715	-0.69957	9.820383795	0.000579911	pumilio homolog 2 [Source:RefSeq peptide;Acc:NP_001100185]
Chrd	A_44_P345319	NM_057134	-0.688721	9.226483843	0.000603483	Chordin [Source:UniProtKB/Swiss-Prot;Acc:Q63148]
Erlin2	A_44_P960610	TC594625	-0.6245076	12.25380353	0.00069046	Erlin-2 [Source:UniProtKB/Swiss-Prot;Acc:B5DEH2]
Agap1	A_43_P10107	NM_001108230	-0.738018	12.45751074	0.000739626	arf-GAP with GTPase, ANK repeat and PH domain-containing protein 1 [Source:RefSeq peptide;Acc:NP_001101700]
Dyrk2	A_44_P411808	NM_001108100	-0.6872124	11.48833158	0.001131427	dual specificity tyrosine-phosphorylation-regulated kinase 2 [Source:RefSeq peptide;Acc:NP_001101570]
COX1	A_44_P183948	ENSRNOT00000043693	0.63358725	14.69525056	0.001321276	cytochrome c oxidase subunit I (mitochondrion) [Source:RefSeq peptide;Acc:YP_665631]
Mat2a	A_44_P118993	NM_134351	-0.6723113	8.767680343	0.001323404	S-adenosylmethionine synthase isoform type-2 [Source:RefSeq peptide;Acc:NP_599178]
Trak2	A_44_P408887	NM_133560	-0.8142061	10.96611248	0.001441981	Trafficking kinesin-binding protein 2 [Source:UniProtKB/Swiss-Prot;Acc:Q8R2H7]

Mmp14	A_43_P14927	NM_031056	-0.9421772	12.79576645	0.001538546	Matrix metalloproteinase-14 [Source:UniProtKB/Swiss-Prot;Acc:Q10739]
Cyr61	A_42_P750683	NM_031327	1.01238313	10.10078298	0.001587414	protein CYR61 precursor [Source:RefSeq peptide;Acc:NP_112617]
Dpt	A_44_P180259	NM_001105965	-0.7300768	11.24557751	0.001856786	dermatopontin precursor [Source:RefSeq peptide;Acc:NP_001099435]
Gcg	A_44_P315672	NM_012707	0.92275337	13.91582294	0.002005436	glucagon preproprotein [Source:RefSeq peptide;Acc:NP_036839]
Tmed9	A_44_P316507	NM_001009703	-0.6923164	13.88545014	0.002190994	Transmembrane emp24 domain-containing protein 9 [Source:UniProtKB/Swiss-Prot;Acc:Q5I0E7]
Lamc1	A_44_P370044	NM_053966	-0.5847798	7.566080452	0.00219839	laminin, gamma 1 precursor [Source:RefSeq peptide;Acc:NP_446418]
Map3k1	A_42_P823894	NM_053887	-0.657362	8.783963992	0.002239734	mitogen-activated protein kinase kinase kinase 1 [Source:RefSeq peptide;Acc:NP_446339]
Sel1l3	A_43_P17569	XM_341223	-0.6296491	12.26811178	0.002253123	Protein Sel1l3 [Source:UniProtKB/TrEMBL;Acc:D3ZIJ4]
Unknown	A_44_P698627	ENSRNOT00000042148	-0.6107699	10.55413705	0.002357382	Unknown
2210407C18Rik	A_44_P375623	NM_001109638	0.60418731	9.734829537	0.002503283	RIKEN cDNA 2210407C18 gene [Source:MGI Symbol;Acc:MGI:1925604]
Otud7b	A_44_P914961	NM_001107697	-1.1844598	7.302714833	0.002510477	OTU domain-containing protein 7B [Source:RefSeq peptide;Acc:NP_001101167]
Tnrc6b	A_44_P484793	NM_138845	-0.7769571	11.63158224	0.0025942	trinucleotide repeat-containing gene 6B protein [Source:RefSeq peptide;Acc:NP_620200]
Chd7	A_42_P690223	ENSRNOT00000008901	-0.8396968	11.7282168	0.002816633	chromodomain helicase DNA binding protein 7 [Source:MGI Symbol;Acc:MGI:2444748]
Plekho2	A_44_P530187	ENSRNOT00000042633	0.67052584	9.604009175	0.002938575	Unknown
Slc28a2	A_44_P523000	XM_001080361	-0.6097043	10.0883756	0.003054584	sodium/nucleoside cotransporter 2 [Source:RefSeq peptide;Acc:NP_113852]
Igf2r	A_44_P1003212	NM_012756	-0.5930957	12.79123785	0.003123251	cation-independent mannose-6-phosphate receptor precursor [Source:RefSeq peptide;Acc:NP_036888]
Mt2A	A_42_P567267	NM_001137564	1.09889944	11.7691076	0.003151135	Metallothionein-2 [Source:UniProtKB/Swiss-Prot;Acc:P04355]
Zfp191	A_44_P301633	NM_182955	-0.659265	11.20369461	0.003352974	Zinc finger protein 24 [Source:UniProtKB/Swiss-Prot;Acc:Q7TNK3]

St6galnac2	A_44_P497302	NM_001031652	-0.6205084	8.509549403	0.003823948	alpha-N-acetylgalactosaminide alpha-2,6-sialyltransferase 2 [Source:RefSeq peptide;Acc:NP_001026822]
Unknown	A_44_P366118	ENSRNOT00000050777	-0.6582971	11.68653514	0.003878182	Unknown
Ppp1r27	A_44_P459858	NM_001105858	0.63979713	7.848837568	0.004434882	dysferlin-interacting protein 1 [Source:RefSeq peptide;Acc:NP_001099328]
Rnf187	A_44_P477268	NM_001164264	0.68226975	9.949953381	0.00455782	E3 ubiquitin-protein ligase RNF187 [Source:RefSeq peptide;Acc:NP_001157736]
Nts	A_44_P1030165	NM_001102381	1.71687516	6.960567984	0.004593693	neurotensin/neuromedin N precursor [Source:RefSeq peptide;Acc:NP_001095851]
Camk2n1	A_44_P493606	NM_173337	-0.8432615	11.85400762	0.004754506	Calcium/calmodulin-dependent protein kinase II inhibitor 1 [Source:UniProtKB/Swiss-Prot;Acc:Q9JI15]
Enpp1	A_43_P12841	NM_053535	-0.8096543	11.19133793	0.004783799	ectonucleotide pyrophosphatase/phosphodiesterase family member 1 [Source:RefSeq peptide;Acc:NP_445987]
Luc7l3	A_44_P424633	NM_001108291	-0.6928149	8.619701732	0.004807296	luc7-like protein 3 [Source:RefSeq peptide;Acc:NP_001101761]
Aldh1a1	A_44_P365286	NM_022407	0.74045003	10.37495918	0.004833248	Retinal dehydrogenase 1 [Source:UniProtKB/Swiss-Prot;Acc:P51647]
Chtf8	A_44_P727274	NM_001194951	0.68312237	10.86914438	0.005173377	Chromosome transmission fidelity protein 8 homolog isoform 2 [Source:UniProtKB/Swiss-Prot;Acc:B2RYL1]
Tcf4	A_42_P524370	NM_053369	-0.6942909	11.45223176	0.005289868	transcription factor 4 [Source:RefSeq peptide;Acc:NP_445821]
Atp2b1	A_43_P12761	NM_053311	-1.2505486	11.58240691	0.005323548	Plasma membrane calcium-transporting ATPase 1 [Source:UniProtKB/Swiss-Prot;Acc:P11505]
Srp72	A_44_P1018684	NM_001170601	-0.7189785	10.42934074	0.005409744	signal recognition particle 72 kDa protein [Source:RefSeq peptide;Acc:NP_001164072]
Irf2bpl	A_42_P491707	FQ226403	-0.6329773	12.12360416	0.005427511	Interferon regulatory factor 2-binding protein-like [Source:UniProtKB/Swiss-Prot;Acc:Q5EIC4]
Napsa	A_42_P738549	NM_031670	-1.9369111	10.46624405	0.005536791	napsin A aspartic peptidase precursor [Source:RefSeq peptide;Acc:NP_113858]
Hyou1	A_43_P11333	NM_138867	-0.8223274	13.02055886	0.005609854	hypoxia up-regulated protein 1 precursor [Source:RefSeq peptide;Acc:NP_620222]
Man2a1	A_44_P1038694	NM_012979	-0.7982794	12.82476989	0.005633241	alpha-mannosidase 2 [Source:RefSeq peptide;Acc:NP_037111]
Mef2bnb	A_44_P468661	NM_001145726	0.66405258	9.48509582	0.005883211	uncharacterized protein LOC688966 [Source:RefSeq peptide;Acc:NP_001139198]
Unknown	A_44_P177801	NM_022397	-0.6247571	12.14096855	0.005953158	Unknown

Igfbp3	A_42_P627998	NM_012588	-1.103182	14.5401056	0.006007802	insulin-like growth factor-binding protein 3 precursor [Source:RefSeq peptide;Acc:NP_036720]
Unknown	A_44_P389664	ENSRNOT00000058640	-0.6806511	12.46474173	0.006269316	Ac2-008 [Source:UniProtKB/TrEMBL;Acc:Q7TPK9]
Akap13	A_44_P210440	NM_001106271	-0.7119876	12.04572814	0.006315607	A-kinase anchor protein 13 [Source:RefSeq peptide;Acc:NP_001099741]
Fam43a	A_44_P356876	NM_001039002	-0.6052845	7.222359874	0.007292567	protein FAM43A [Source:RefSeq peptide;Acc:NP_001034091]
Cyp4v3	A_44_P297252	NM_001135600	-0.8064656	7.27739549	0.007323899	Cytochrome P450 4V2 [Source:UniProtKB/Swiss-Prot;Acc:A2RRT9]
Tspan12	A_42_P665848	NM_001015026	-0.5982725	8.993980367	0.0077037	Tetraspanin-12 [Source:UniProtKB/Swiss-Prot;Acc:Q569A2]
Unknown	A_44_P898364	ENSRNOT00000033720	0.69364771	8.949627744	0.007881846	Uncharacterized protein [Source:UniProtKB/TrEMBL;Acc:F1LXN7]
Eif4ebp2	A_44_P757980	NM_001033069	-0.9809898	11.56735852	0.008031728	eukaryotic translation initiation factor 4E-binding protein 2 [Source:RefSeq peptide;Acc:NP_001028241]
Adh7	A_44_P166161	NM_134329	0.71149299	7.543402392	0.008308359	alcohol dehydrogenase class 4 mu/sigma chain [Source:RefSeq peptide;Acc:NP_599156]
Elovl6	A_44_P111489	TC614535	-0.6129884	9.631538258	0.008579145	elongation of very long chain fatty acids protein 6 [Source:RefSeq peptide;Acc:NP_599210]
Egfr	A_44_P346408	NM_031507	-0.6210015	9.090549677	0.008586236	epidermal growth factor receptor precursor [Source:RefSeq peptide;Acc:NP_113695]
Eif4e3	A_44_P466595	NM_001106612	-0.653951	9.017503424	0.008921299	eukaryotic translation initiation factor 4E type 3 [Source:RefSeq peptide;Acc:NP_001100082]
Rtn4rl2	A_44_P203173	NM_181380	0.95656756	11.56492693	0.008942998	Reticulon-4 receptor-like 2 [Source:UniProtKB/Swiss-Prot;Acc:Q80WD1]
Myocd	A_44_P479899	NM_182667	-0.6127353	7.732218582	0.008955701	Myocardin [Source:UniProtKB/Swiss-Prot;Acc:Q8R5I7]
Unknown	A_44_P288536	NM_001108548	0.61398757	10.51205816	0.009901751	Unknown

Supplementary Figure S1. Cerebral Network view of significantly over-represented gene ontology biological processes, KEGG pathways and Reactome pathways among differentially expressed genes in the colon of germ-free rats mono-associated with AGR1485 or AGR1487. Network shows processes and pathways linked by shared genes, arranged according to cellular localization.

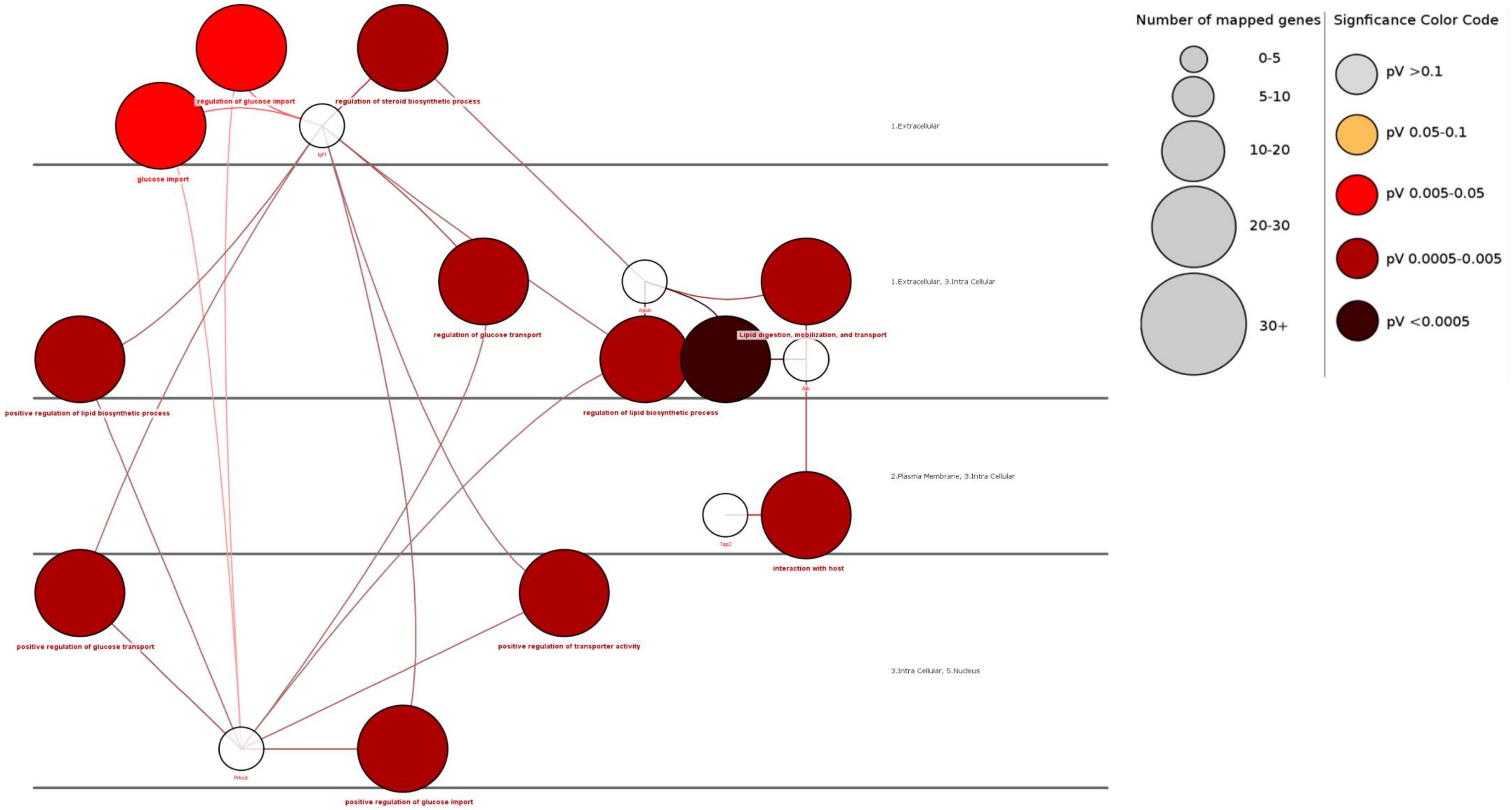

Supplement: Supplementary Information [file srep20318-s1.pdf]
